# Delivering a drug study in primary care: trial management challenges and solutions

**DOI:** 10.1186/1745-6215-16-S2-P179

**Published:** 2015-11-16

**Authors:** Seonaidh Cotton, Karen Innes, David Price, Graham Devereux

**Affiliations:** 1 University of Aberdeen, Aberdeen, UK

## 

The NIHR HTA funded TWICS trial is investigating whether low-dose theophylline (as an adjunct to existing inhaled corticosteroids) reduces exacerbations in patients with Chronic Obstructive Pulmonary Disease. It is a pragmatic, double-blind, randomised controlled trial aiming to recruit 1424 participants; with at least 50% from primary care. The trial was therefore designed to be delivered in both primary and secondary care settings. We will present some of the challenges that we have faced, together with the solutions we have implemented: two of these are described below.

There are challenges around delivery of the intervention. Over the 12 month follow-up, participants receive 13 bottles of once or twice daily study medication. For participants recruited in secondary care sites, the first of these bottles is dispensed from the site's clinical trials pharmacy; for those recruited in primary care, it is dispensed from the lead site's clinical trials pharmacy and couriered to their home. For all participants, all subsequent supplies of study medication are couriered direct to their home. We have now recruited 650 participants, all of whom have received their supplies of study medication.

There are also challenges around training and initiation of multiple primary care sites, which are needed to meet the primary care recruitment target. We have adopted a “train the trainer” model and have a network of research nurses who can deliver study training to primary care practices across the North and East of England. To date, they have trained > 75 primary care practices to deliver the study.

